# Effect of Point-Spread Function Reconstruction for Indeterminate PSMA-RADS-3A Lesions on PSMA-Targeted PET Imaging of Men with Prostate Cancer

**DOI:** 10.3390/diagnostics11040665

**Published:** 2021-04-07

**Authors:** Wajahat Khatri, Hyun Woo Chung, Rudolf A. Werner, Jeffrey P. Leal, Kenneth J. Pienta, Martin A. Lodge, Michael A. Gorin, Martin G. Pomper, Steven P. Rowe

**Affiliations:** 1 The Russell H. Morgan Department of Radiology and Radiological Science, Johns Hopkins University School of Medicine, Baltimore, MD 21287, USA; wkhatri1@jhmi.edu (W.K.); jleal1@jhmi.edu (J.P.L.); mlodge1@jhmi.edu (M.A.L.); mpomper@jhmi.edu (M.G.P.); 2 Department of Nuclear Medicine, Konkuk University Medical Center, Konkuk University School of Medicine, Seoul 05030, Korea; 20080193@kuh.ac.kr; 3 Department of Nuclear Medicine, University Hospital Würzburg, 97080 Würzburg, Germany; Werner_R1@ukw.de; 4 The James Buchanan Brady Urological Institute and Department of Urology, Johns Hopkins University School of Medicine, Baltimore, MD 21287, USA; kpienta1@jhmi.edu; 5 Department of Urology, University of Pittsburgh School of Medicine, Pittsburgh, PA 15232, USA; michaelagorin@gmail.com; 6 Urology Associates and UPMC Western Maryland, Cumberland, MD 21502, USA

**Keywords:** prostate-specific membrane antigen, reporting and data system, positron emission tomography

## Abstract

Purpose: Prostate-specific membrane antigen (PSMA) positron emission tomography (PET) is emerging as an important modality for imaging patients with prostate cancer (PCa). As with any imaging modality, indeterminate findings will arise. The PSMA reporting and data system (PSMA-RADS) version 1.0 codifies indeterminate soft tissue findings with the PSMA-RADS-3A moniker. We investigated the role of point-spread function (PSF) reconstructions on categorization of PSMA-RADS-3A lesions. Methods: This was a post hoc analysis of an institutional review board approved prospective trial. Around 60 min after the administration of 333 MBq (9 mCi) of PSMA-targeted ^18^F-DCFPyL, patients underwent PET/computed tomography (CT) acquisitions from the mid-thighs to the skull vertex. The PET data were reconstructed with and without PSF. Scans were categorized according to PSMA-RADS version 1.0, and all PSMA-RADS-3A lesions on non-PSF images were re-evaluated to determine if any could be re-categorized as PSMA-RADS-4. The maximum standardized uptake values (SUVs) of the lesions, mean SUVs of blood pool, and the ratios of those values were determined. Results: A total of 171 PSMA-RADS-3A lesions were identified in 30 patients for whom both PSF reconstructions and cross-sectional imaging follow-up were available. A total of 13/171 (7.6%) were re-categorized as PSMA-RADS-4 lesions with PSF reconstructions. A total of 112/171 (65.5%) were found on follow-up to be true positive for PCa, with all 13 of the re-categorized lesions being true positive on follow-up. The lesions that were re-categorized trended towards having higher SUV_max_-lesion and SUV_max_-lesion/SUV_mean_-blood-pool metrics, although these relationships were not statistically significant. Conclusions: The use of PSF reconstructions for ^18^F-DCFPyL PET can allow the appropriate re-categorization of a small number of indeterminate PSMA-RADS-3A soft tissue lesions as more definitive PSMA-RADS-4 lesions. The routine use of PSF reconstructions for PSMA-targeted PET may be of value at those sites that utilize this technology.

## 1. Introduction

Prostate cancer (PCa) is the most common noncutaneous malignancy in men as well as the second most common cause of cancer death in men in the U.S.A. [1]. The high incidence of PCa has been a driving force for the development of new imaging techniques to improve staging and re-staging. Although conventional imaging with computed tomography (CT) and bone scan can adequately stage patients with widespread metastatic disease, the introduction of radiopharmaceuticals that bind the prostate-specific membrane antigen (PSMA), for targeted positron emission tomography/computed tomography (PET/CT), has facilitated the detection of small-volume lesions that can significantly alter treatment planning [2].

PSMA is a class II transmembrane glycoprotein that is a valuable target for imaging and therapy, as it is significantly overexpressed on malignant prostate cells [3]. Among the emerging indications for PSMA PET is the evaluation of patients with biochemical recurrence (BCR) after initial curative-intent therapy [4] and the identification of men with oligometastatic PCa (typically being defined as ≤5 sites of distant disease) [5]. Sensitive lesion detection in men with BCR or oligometastatic disease is desired for directing further treatment, which might include targeted radiotherapy or surgery [6].

Given the increased use of such therapies for limited volume recurrent/metastatic disease, the confident characterization of lesions on PSMA PET is an important clinical need. For this reason, we developed the PSMA-RADS version 1.0 scoring system, which includes indeterminate categories for lesions that cannot be definitively ascertained to be PCa [7,8,9,10]. One such category is PSMA-RADS-3A, which describes lymph nodes and other soft tissue lesions that would be in the typical distribution of PCa but are small and have low-level uptake. We have previously found that approximately 75% of PSMA-RADS-3A lesions will manifest as true positive sites of PCa on follow-up imaging [9]. Improvements in the evaluation of these small and generally subtle lesions so that they could be more definitively assigned as benign or malignant findings would be of value to guide accurate therapy planning.

Modern image reconstruction techniques, including point-spread function (PSF) reconstruction, can lead to improved signal-to-noise ratio and conspicuity of small or subtle lesions [11]. Hence, the aim of this study was to evaluate if PSF reconstructions for ^18^F-DCFPyL PET/CT affect the characterization of PSMA-RADS-3A lesions.

## 2. Materials and Methods

### 2.1. Patient Population

Patients with a history of pathologically diagnosed PCa who had undergone an ^18^F-DCFPyL PET/CT scan on an institutional review board approved prospective research protocol (ClinicalTrials.gov (accessed on 1 January 2021) NCT02825875) were screened as part of this post hoc analysis to identify patients with at least one PSMA-RADS-3A lesion. All patients had signed written, informed consent to participate in the original research trial. Patients were imaged under the auspices of a US Food and Drug Administration Investigational New Drug Application (IND 121064). Clinical and demographic information, including age, serum prostate-specific antigen (PSA) levels, and PCa treatment history, was collected.

### 2.2. Image Acquisition

The radiosynthesis of ^18^F-DCFPyL was performed as previously described [12]. Images were acquired in a manner consistent with prior studies [13]. In brief, all patients were asked to refrain from eating or drinking (other than water and medications) for at least 4 h before the intravenous injection of approximately 333 MBq (9 mCi) of ^18^F-DCFPyL. One hour after the injection, whole-body PET/CT was performed (from the midthighs to the vertex of the skull) on one of two clinical scanners. Patients included in the final analysis were imaged on a 128-slice Biograph mCT scanner (Siemens) and reconstructed with both a standard ordered subset expectation maximization (OSEM) algorithm and OSEM with PSF. The standard reconstruction algorithm involved 3D OSEM with time-of-flight, 2 iterations, 21 subsets, and a 5 mm Gaussian filter. The higher spatial resolution PSF reconstruction involved 3D OSEM with time-of-flight and PSF modelling, 2 iterations, 21 subsets, and no post-reconstruction filter. CT (40 effective mAs) was utilized for attenuation correction.

### 2.3. Image Analysis

^18^F-DCFPyl PET/CT scans reconstructed with OSEM but without PSF were centrally reviewed by an experienced reader (HWC), and lesions were characterized according to PSMA-RADS version 1.0. A second experienced reader (SPR) verified the lesion characterizations on OSEM without PSF and then re-characterized the PSMA-RADS-3A lesions with OSEM with PSF. As defined in the original description of the PSMA-RADS 1.0 scoring system, the central reviewers considered PSMA-RADS-3A lesions to be lymph node or soft tissue findings that had subtle radiotracer uptake (approximately blood pool or slightly higher) and that were in a typical pattern of distribution for PCa, such as the pelvis and retroperitoneum [7,8]. Additionally, radiotracer uptake in the mediastinum and left supraclavicular space in patients with more advanced disease was considered as typical sites of PCa spread. Lesions that appeared more conspicuous with PSF were re-categorized as PSMA-RADS-4: a category that signifies distinct uptake in un-enlarged lymph nodes or other morphologically normal soft tissue structures and that is highly suggestive of the presence of PCa.

For those patients for whom it was available, follow-up cross-sectional imaging was reviewed in order to establish which PSMA-RADS-3A lesions developed into true positive sites of disease. No specific limitations were set on the type of follow-up imaging that could be used, and imaging included repeat ^18^F-DCFPyL PET/CT, ^18^F-fluciclovine PET/CT, CT, or MRI. Lesions that were determined on follow-up to be suggestive of the presence of PCa met at least one of the following criteria, adapted from [9]:

1. Follow-up PET/CT imaging with ^18^F-DCFPyL with significantly increasing or decreasing uptake (defined as a change of 30% from baseline) after therapy OR significantly increased uptake during observation. If the follow-up PET/CT was performed with ^18^F-fluciclovine, focal uptake in a lesion in concordance with the read paradigm [14] for that agent was taken as evidence of true positivity.
2. Anatomic imaging with CT or MRI demonstrating a ≥2 mm increase in the long axis diameter of the lesion during a period of observation or a decrease in the lesion long axis diameter of ≥2 mm after beginning PCa treatment.

In addition to PSMA-RADS version 1.0 categorization, the SUV_max_ corrected for lean body mass for all of the lesions and the SUV_mean_ of blood pool (determined by a 3 cm sphere in the ascending aorta) were measured. The ratios of SUV_max_ of each lesion corrected for the SUV_mean_ of blood pool (SUV_max_-lesion/SUV_mean_-blood-pool) were calculated.

### 2.4. Statistical Analysis

Descriptive statistics were utilized for this post hoc analysis. Patient demographic and clinical information are given as medians with ranges or percentages, as appropriate. SUV_max_, SUV_mean_, and SUV_max_lesion/SUV_mean_-blood-pool are expressed as medians with interquartile ranges (IQRs). Median values were compared using the Mann–Whitney U test with *p*-values < 0.05 considered significant. Statistical analysis was performed with PASW Statistics 17.0 (Chicago, IL, USA).

## 3. Results

### 3.1. Patients

Clinical and imaging parameters of 275 patients were screened. Of those, 89 patients were categorized as having at least one PSMA-RADS-3A lesion on standard clinical reconstructions of their ^18^F-DCFPyL PET/CT scans. Thirty of those patients were found to (1) have been imaged on the Siemens Biograph mCT scanner with PSF reconstruction images of their ^18^F-DCPFyL PET/CTs available in the picture archiving and communication system (PACS) and (2) had adequate follow-up cross-sectional imaging to evaluate for potential changes over time in the PSMA-RADS-3A lesions.

Those 30 patients were included in the presented analysis. Their median age was 72.5 (range 59–81) and median serum PSA at the time of imaging was 3.8 (range 0.2–43.9). Twenty-two (73.3%) underwent imaging for biochemical recurrence or PSA persistence following local therapy, 4 (13.3%) underwent imaging for initial PCa staging, and 4 (13.3%) underwent imaging for evaluation of metastatic disease. With regard to available follow-up imaging, 20 (66.7%) underwent diagnostic abdomen/pelvis CT scans, 6 (20.0%) underwent repeat ^18^F-DCFPyL PET/CT, 3 (10.0%) underwent ^18^F-fluciclovine PET/CT, and 1 (3.3%) underwent a pelvic MRI. Follow-up imaging was performed at a median of 11 months (range: 2–36 months) post initial ^18^F-DCFPyL PET/CT. Selected demographic and clinical information of the study cohort are included in Table 1.

### 3.2. Image Analysis

**Figure 1:**
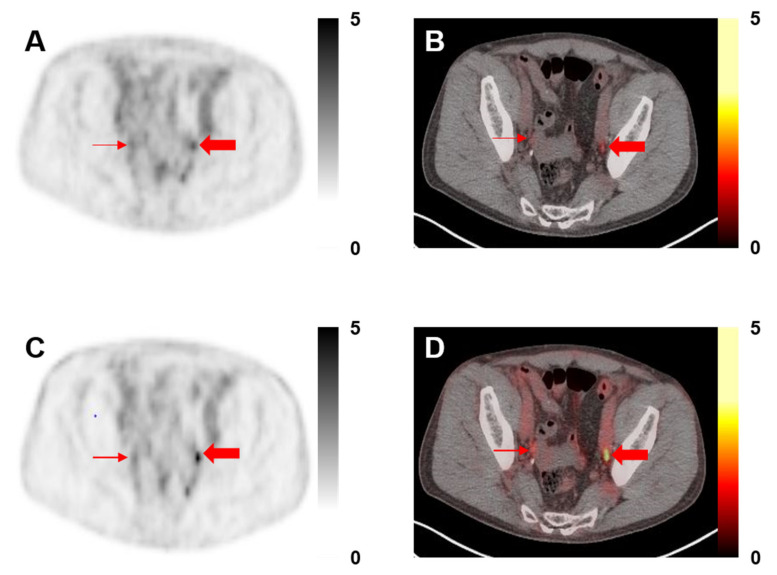
A 68-year-old man with history of prostatectomy and adjuvant chemohormonal therapy, now with an increase in prostate-specific antigen (PSA) to 0.3. (**A**) ^18^F-DCFPyL positron emission tomography (PET) and (**B**) PET/computed tomography (CT) axial images without point-spread function (PSF) demonstrating bilateral PSMA-RADS-3A pelvic lymph nodes (thin and thick red arrows, maximum standardized uptake value (SUV_max_) 1.2 on the right and 1.5 on the left). (**C**) ^18^F-DCFPyL PET and (**D**) PET/CT axial images with PSF show that the right pelvic PSMA-RADS-3A lymph node does not appear significantly more conspicuous (SUV_max_ 2.2) and that lesion was not re-categorized. However, the lymph node on the left (SUV_max_ 2.5) was thought to be more conspicuous and was re-categorized to PSMA-RADS-4.

Among the 30 patients included in this study, a total of 171 lesions were designated as PSMA-RADS-3A (median 4 per patient, range 1–23), all of which were lymph nodes. Of these lesions, 13/171 (7.6%) demonstrated visual changes with PSF reconstructions such that they were re-categorized as PSMA-RADS-4 (Figure 1). A total of 112/171 (65.5%) lesions were found on follow-up imaging to be indicative of PCa, 32/112 (28.6%) by category 1 above, and 80/112 (71.4%) by category 2 above. A total of 13/13 (100.0%) of the re-categorized lesions were found to be true positive on follow-up imaging, whereas 99/158 (62.7%) lesions that were still considered indeterminate on the PSF reconstructions were found to be true positive on follow-up.

**Figure 2:**
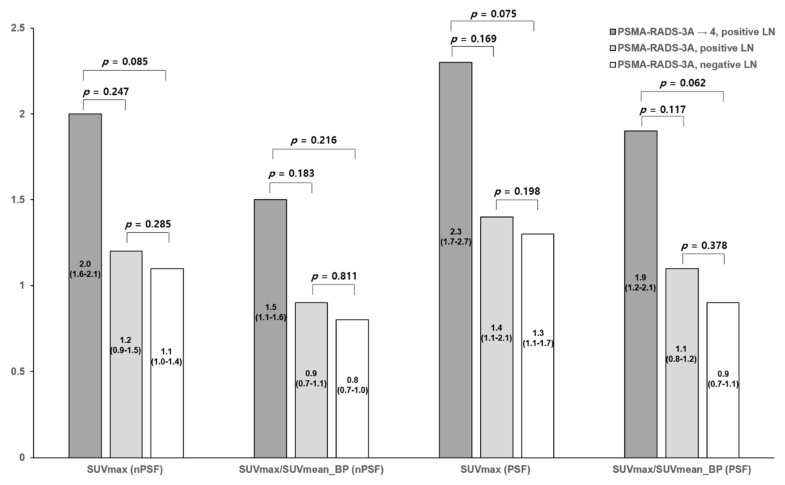
Bar graph representation of medians and interquartile ranges for the SUV_max_-lesion and SUV_max_-lesion/SUV_mean_-blood-pool metrics of the PSMA-RADS-3A lesions included in this study. In each bar of the graph, the top number is the median and the interquartile ranges are listed below. Note the trends towards higher SUVs of the lesions that were re-categorized as PSMA-RADS-4 (dark grey), although none of these relationships reached statistical significance (i.e., all *p* > 0.05). SUV = standardized uptake value; nPSF = non-point-spread function; BP = blood-pool; PSF = point-spread function; LN = lymph node.

**Figure 3:**
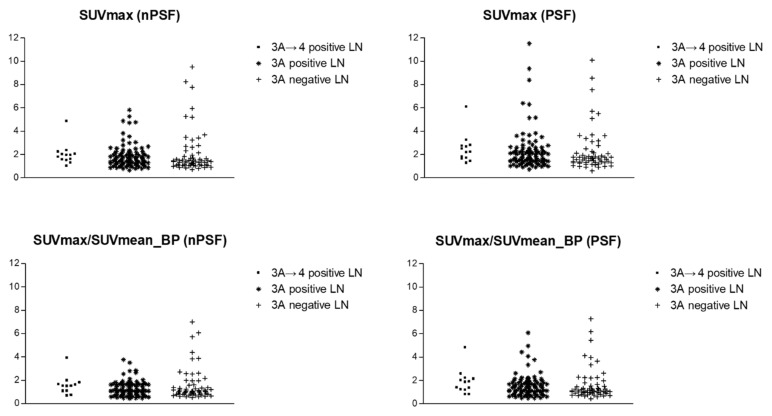
Distributions of SUV_max_-lesion and SUV_max_-lesion/SUV_mean_-blood-pool for the different categories of lymph nodes included in this study. The outlier high uptake values were related to overlap of other high uptake structures in the manual segmentations of the lesions. SUV = standardized uptake value; nPSF = non-point-spread function; BP = blood-pool; PSF = point-spread function; LN = lymph node.

Uptake characteristics of lesions are summarized in Figure 2 and Figure 3. The analyses based on SUV_max_-lesion and SUV_mean_-blood-pool demonstrated that SUVs were generally higher on PSF reconstructed images. The SUV_max_-lesion median value for all lesions on non-PSF images was 1.7 (IQR 1.2–2.1). The SUV_max_-lesion median value all lesions on PSF images was 1.8 (IQR 1.4–2.5). The SUV_mean_-blood-pool median on non-PSF images was 1.4 (IQR 1.3–1.5) and on PSF images was 1.4 (IQR 1.3–1.6). SUV_max_-lesion/SUV_mean_-blood-pool median for all lesions on non-PSF images was 1.1 (IQR 0.8–1.6). SUV_max_-lesion/SUV_mean_-blood-pool for all lesions on PSF images was 1.3 (IQR 0.9–1.9).

For the 13 PSMA-RADS-3A lesions that were re-categorized to PSMA-RADS-4, the SUV_max_-lesion and SUV_max_-lesion/SUV_mean_-blood-pool on both non-PSF and PSF images were generally higher than those lesions that were not re-categorized (Figure 2), although these differences were not statistically significant (*p* = 0.085–0.216). Similar to our prior findings [9], there was no difference in the SUV_max_-lesion and SUV_max_-lesion/SUV_mean_-blood-pool values between those lesions that were true positive and those that were not confirmed to be true positive (Figure 2, *p* = 0.198–0.811). The distributions of uptake in the different lesion categories overlapped on both non-PSF and PSF reconstructed images (Figure 3).

## 4. Discussion

PSMA-targeted PET has been rapidly adopted around the world for PCa imaging given its high sensitivity and specificity for the identification of sites of disease [15,16]. However, as with any imaging modality, there are indeterminate findings that belie easy categorization [17]. The preponderance of studies on PSMA-targeted PET imaging has been retrospective, further hindering the ability of readers to know how best to guide management of indeterminate findings, although large prospective studies are starting to appear in the literature [18].

In this study, PSF reconstruction methods for ^18^F-DCFPyL PET/CT scans were evaluated in terms of characterization of indeterminate PSMA-RADS-3A lesions. We found that PSF reconstructions can increase the contrast and activity concentration within PSMA-RADS-3A lesions, and that based on a visual analysis, there was a subset of lesions that were changed to the more definitive category of PSMA-RADS-4 (7.6%). Of those lesions that were determined to change to PSMA-RADS-4 on the PSF-reconstructed images, all were determined to be true positive on follow-up imaging. This suggests that PSF reconstructions can increase confidence in the characterization of a small number of indeterminate lesions on PSMA-targeted PET scans, and that confidence does not come at the cost of decreased specificity. It should be noted that the lesions that were re-categorized tended to have higher SUV_max_-lesion and SUV_max_-lesion/SUV_mean_-blood-pool than those lesions that were not re-categorized (although the trend did not reach significance), suggesting that careful segmentation and determination of semi-quantitative parameters may aid in PSMA-RADS classification and that it may not be optimized as a purely visual method of lesion categorization.

Regardless of reconstruction methodology, the vast majority of indeterminate soft tissue lesions on PSMA PET will remain indeterminate. As we have previously shown [9], and consistent with the present work, a sizable majority of indeterminate soft tissue lesions will be true positive on follow-up (65.5% in this study, similar to the 75.0% reported in [9]), and it may be prudent to include such lesions in the planning of salvage therapies, such as stereotactic body radiation therapy for oligometastatic disease [19]. A subset of lesions that could not be confirmed as true positive may still represent sites of PCa; however, they did not undergo the changes that we had designated a priori as indicative of true positive lesions. It is likely that artificial intelligence algorithms will be able to leverage the large amounts of scan data to eventually aid in the definitive characterization of otherwise indeterminate findings [20].

An important limitation of this study is the lack of histopathologic confirmation of PSMA-RADS-3A lesions. Such lesions on ^18^F-DCFPyL PET scans are often smaller than 1 cm, making biopsy impossible to perform. Thus, we were required to rely upon imaging follow-up to define a true positive lesion. Other limitations of our study include its post hoc design and small sample size of only 30 patients. Nonetheless, a large number of lesions were analyzed allowing for confidence in our findings. Further, the central review that was utilized was primarily driven by one reader with a second, corroborating reader; this could have introduced bias. Lastly, for small lesions with subtle uptake, the physical differences in image quality between ^18^F and ^68^Ga may be relevant, and it is uncertain if the findings in this study would directly apply to ^68^Ga-labeled PSMA agents. Certainly, given these limitations, further study of this topic is warranted.

## 5. Conclusions

We evaluated the impact of PSF reconstruction for ^18^F-DCFPyL PET/CT on indeterminate PSMA-RADS-3A lesions in patients with PCa. A small number of lesions became more definitively characterized with the addition of PSF, and all of those that were re-categorized were found to be true positive on follow-up. We conclude that PSF reconstructions for PSMA-targeted PET may appropriately increase confidence in the characterization of a small, yet clinically significant, number of otherwise indeterminate lesions.

## Figures and Tables

**Table 1:** Treatment data from patients included in this study.

Therapy	Percent
Pre-Scan Therapy	
- Prostatectomy	70%
- Radiation	36%
- Salvage Radiation	30%
- Brachytherapy	3%
- Cryoablation	3%
- ADT	30%
- Salvage PLND	3%
- None	13%
Post-Scan Therapy	
- Prostatectomy	7%
- Cryoablation	3%
- Radiation	23%
- ADT	60%
- Salvage PLND	7%
- Chemotherapy	20%
- Observation	13%

Abbreviations: ADT = androgen deprivation therapy; PLND = pelvic lymph node dissection.

## Data Availability

The data presented in this study are available on request from the corresponding author. The data are not publicly available due to privacy limitations.
